# Anti-Tumor Potential of IMP Dehydrogenase Inhibitors: A Century-Long Story

**DOI:** 10.3390/cancers11091346

**Published:** 2019-09-11

**Authors:** Rand Naffouje, Punita Grover, Hongyang Yu, Arun Sendilnathan, Kara Wolfe, Nazanin Majd, Eric P. Smith, Koh Takeuchi, Toshiya Senda, Satoshi Kofuji, Atsuo T. Sasaki

**Affiliations:** 1 Division of Hematology and Oncology, Department of Internal Medicine, University of Cincinnati College of Medicine, Cincinnati, OH 45267, USA; naffourd@ucmail.uc.edu (R.N.); groverpt@ucmail.uc.edu (P.G.); arunsendil@gmail.com (A.S.);; 2 Structural Biology Research Center, Institute of Materials Structure Science, High Energy Accelerator Research Organization (KEK), Tokyo 135-0063, Japan; yuhong@post.kek.jp (H.Y.); toshiya.senda@kek.jp (T.S.); 3 Department of Accelerator Science, School of High Energy Accelerator Science, SOKENDAI (the Graduate University for Advanced Studies), 1-1 Oho, Tsukuba, Ibaraki 305-0801, Japan; 4 Department of Cancer Biology, University of Cincinnati College of Medicine, Cincinnati, OH 45267, USA; 5 Department of Neuro-oncology, The University of Texas MD Anderson Cancer Center, 1515 Holcombe Blvd., Houston, TX 77030, USA; nkmajd@mdanderson.org; 6 Department of Internal Medicine, University of Cincinnati College of Medicine, Cincinnati, OH 45267, USA; smithep@ucmail.uc.edu; 7 Molecular Profiling Research Center for Drug Discovery, National Institute of Advanced Science and Technology, 2-3-26 Aomi, Koto, Tokyo 135-0063, Japan; koh-takeuchi@aist.go.jp; 8 Graduate School of Biomedical & Health Sciences, Hiroshima University, Hiroshima 734-8553, Japan; sakofuji@hiroshima-u.ac.jp; 9 Department of Neurosurgery, Brain Tumor Center at UC Gardner Neuroscience Institute, Cincinnati, OH 45267, USA; 10 Institute for Advanced Biosciences, Keio University, Tsuruoka, Yamagata 997-0052, Japan

**Keywords:** IMPDH, IMPDH inhibitors, mycophenolic acid, GTP, purine synthesis, anti-tumor

## Abstract

The purine nucleotides ATP and GTP are essential precursors to DNA and RNA synthesis and fundamental for energy metabolism. Although de novo purine nucleotide biosynthesis is increased in highly proliferating cells, such as malignant tumors, it is not clear if this is merely a secondary manifestation of increased cell proliferation. Suggestive of a direct causative effect includes evidence that, in some cancer types, the rate-limiting enzyme in de novo GTP biosynthesis, inosine monophosphate dehydrogenase (IMPDH), is upregulated and that the IMPDH inhibitor, mycophenolic acid (MPA), possesses anti-tumor activity. However, historically, enthusiasm for employing IMPDH inhibitors in cancer treatment has been mitigated by their adverse effects at high treatment doses and variable response. Recent advances in our understanding of the mechanistic role of IMPDH in tumorigenesis and cancer progression, as well as the development of IMPDH inhibitors with selective actions on GTP synthesis, have prompted a reappraisal of targeting this enzyme for anti-cancer treatment. In this review, we summarize the history of IMPDH inhibitors, the development of new inhibitors as anti-cancer drugs, and future directions and strategies to overcome existing challenges.

## 1. Introduction

Purine nucleotides (e.g., ATP and GTP) are involved in many cellular functions including serving as building blocks for DNA and RNA, sources of energy, enzyme cofactors in metabolic pathways, and components of signal transduction. More specifically, GTP is a purine nucleoside triphosphate used as a source of energy for protein synthesis and a signaling molecule that regulates various cellular processes. Cellular GTP concentrations are markedly elevated in many types of cancers [1,2]. Until recently, the upregulation of the GTP pool size in cancers was thought likely to be an epiphenomenon. However, recently, we have shown that this is a primary result of elevated GTP synthesis via upregulation of the rate-limiting enzyme of the de novo GTP nucleotide synthesis pathway, known as inosine monophosphate dehydrogenase (IMPDH) [2]. Moreover, this increased synthesis directly increases cellular anabolism and induces malignant transformation of tumors.

The human genome encodes two IMPDH isoenzymes, *IMPDH1* on chromosome 7 and *IMPDH2* on chromosome 3. Unlike IMPDH1, studies suggest that *IMPDH2* expression is elevated in neoplastic cells [3,4,5]. We and others recently reported the importance of the GTP de novo pathway in glioblastoma [2], brain tumor initiating cells [6], mTORC1-activated tumors [7], and a subset of small cell lung cancers [8]. These findings suggest de novo guanine nucleotide biosynthesis through IMPDH may be a promising therapeutic target for some cancers. Mycophenolic acid (MPA), the first IMPDH inhibitor discovered more than 100 years ago, has shown anti-tumor activity in various cancer cell lines and mouse models [9,10,11]. However, despite these long-known anti-tumor actions, no IMPDH inhibitor has been clinically approved as an anti-cancer drug in large part due to side effects at high treatment dose and variable responses. In this paper, we will review the history of IMPDH inhibitors, the reasons for the limited progress in establishing an effective antitumor derivative, and the prospects for successful development of new inhibitors.

## 2. Historical Review of IMPDH Inhibitors: The Discovery of Mycophenolic Acid

**Figure 1:**
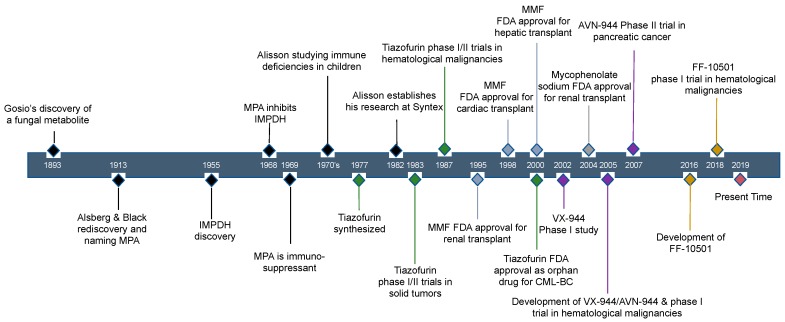
Timeline of events in the history of MPA and other IMPDH inhibitors.

The history of the first IMPDH inhibitor, mycophenolic acid (MPA), dates back more than 100 years ago with its purification of penicillium fungal species culture in 1893 by the Italian scientist, Dr. Bartolomio Gosio (Figure 1). He was searching for the etiology of pellagra in populations in which corn is a primary dietary staple. In that era, before the discovery that pellagra was caused by the lack of niacin (vitamin B3), pellagra was speculated to be secondary to toxin-producing bacteria or fungus in spoiled corn [12]. Although Dr. Gosio did not elucidate the cause of pellagra, he identified a fungal metabolite that inhibited the growth of *Bacillus anthracis* [13].

In 1913, two American Department of Agriculture chemical biologists, Drs. Alsberg and Black, reassessed this metabolite [14] during a pellagra epidemic in the US. Since they detected the presence of the active metabolite in spoiled corn, which rendered acidic properties by fungal infections, they chose to name the compound mycophenolic acid denoting an acidic phenol from a fungus (prefix “myco-“ means fungus) observing, at the time, the close similarity to the compound purified by Dr. Gosio 20 years earlier. In 1928, Dr. Alexander Fleming noticed the antibacterial effects of contaminating mold in petri dish cultures of *Staphylococcus,* but actual purification and use of penicillin as an antibiotic was not achieved until 1939. While penicillin is traditionally considered to be the first true antibiotic, in reality, MPA, purified in 1893 by Dr. Gosio, could be considered to be the first antibiotic. However, MPA was abandoned as a feasible antibiotic, partly due to its gastrointestinal toxicity at effective doses [15].

## 3. MPA Inhibits IMPDH Activity and Possesses an Immunosuppressive Effect

**Figure 2:**
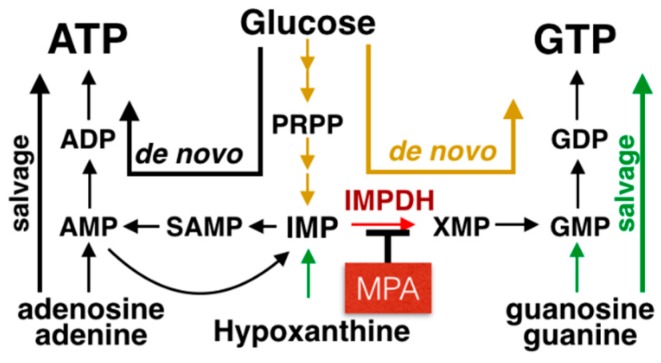
MPA: Mechanism of Action. IMPDH catalyzes the rate-limiting, NAD-dependent oxidation of inosine monophosphate (IMP) to xanthosine 5′-monophosphate (XMP), which is an intermediate metabolite in the production of guanosine-triphosphate (GTP). MPA is a potent, selective, reversible, and noncompetitive inhibitor of IMPDH. Abbreviations: SAMP: succinyl-AMP, and PRPP: phosphoribosyl pyrophosphate.

In 1955, IMPDH was first described in the investigation of purine biosynthesis as a NAD^+^ requiring dehydrogenase necessary to convert inosine monophosphate (IMP) to xanthosine monophosphate (XMP) in rabbit bone marrow extracts [16,17] and pigeon liver extracts [18]. IMPDH is the rate-limiting step in de novo biosynthesis of guanine nucleotides (Figure 2). The fundamental observation that MPA inhibits IMPDH was first reported in the UK in 1968 in a patent application (application no. 26562/68), but the complete specification was reported in 1969 [19,20].

**Figure 3:**
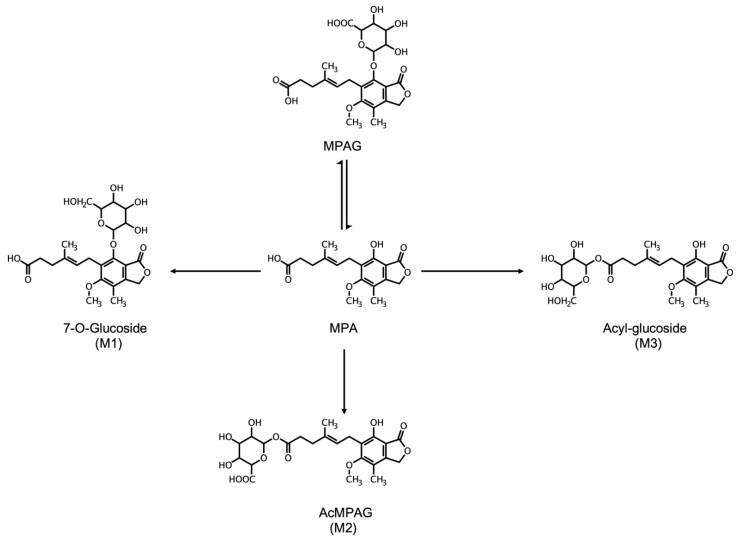
MPA and its metabolites: phenolic MPA-glucuronide (MPAG), phenolic 7-0-glucoside (M1), acyl glucuronide (M2), and acyl-glucoside (M3), a CYP450 oxidation product.

In the 1970s, at the Medical Research Council’s Clinical Research Center in London, the South African geneticist, Dr. Anthony Allisson, was investigating biochemical causes of immune deficiency disorders in children. He discovered that the defect of adenosine deaminase (ADA) in the patient was accompanied by decreased guanine nucleotides [21]. Coincidently, in 1969, a Japanese group primarily investigating the antibiotic effects of MPA [22] reported immunosuppressant properties of MPA. Dr. Allison predicted that depleting pools of GTP would have immunosuppressive effects on lymphocytes and set out to improve the oral bioavailability of MPA. This led to the development of a pro-drug amino ester derivative of MPA, mycophenolate mofetil (MMF), which was activated after de-esterification by the liver [23] (Figure 3). After it underwent large successful clinical trials, MMF was approved by the U.S. Food and Drug Administration (FDA) in 1995 as an immunosuppressant drug for use in solid organ transplantation and was marketed under the brand CellCept (Roche). In 2004, an enteric-coated formulation of mycophenolic acid (mycophenolate sodium) was also approved as an immunosuppressant in organ transplant and marketed as Myfortic (Novartis). Table 1 summarizes labeled (FDA approved) and off-label indications of MMF.

## 4. Current Use of IMPDH Inhibitors and Their Application in Cancer Therapy

The development of IMPDH inhibitors as anti-cancer drugs can be divided into three eras. The first era was highlighted by the appreciation in the 1960s and 1970s of the anti-tumor activity of MPA, and these effects were discovered in the context of burgeoning interest in GTP metabolism in many overlapping fields. Notably, MPA was shown to have antiviral [59], antifungal [60], antibacterial [60], antitumor [9,59], anti-psoriasis [61], and immunosuppressant properties [22]. Specifically, with respect to cancer, MPA was shown to have anti-tumor effects in cell lines obtained from different malignancies and murine models (see Section 4.1). Based on these early preclinical studies, a second era (1980s–2000s) expanded the focus to a variety of potential clinical applications. MMF and competitive inhibitors such as tiazofurin and small molecule non-competitive inhibitors like VX-944 were developed. However, phase II cancer trials showed limited clinical efficacy. The third and current era (2015–present) was propelled by advances in molecular analyses (e.g., CRISPR/Cas9-based gene manipulation, mass spectrometry-based metabolome), which lead to a renewed interest in the anti-cancer potential of IMPDH inhibitors.

### 4.1. Evidence of the Antitumor Activity of IMPDH Inhibitors

**Figure 4:**
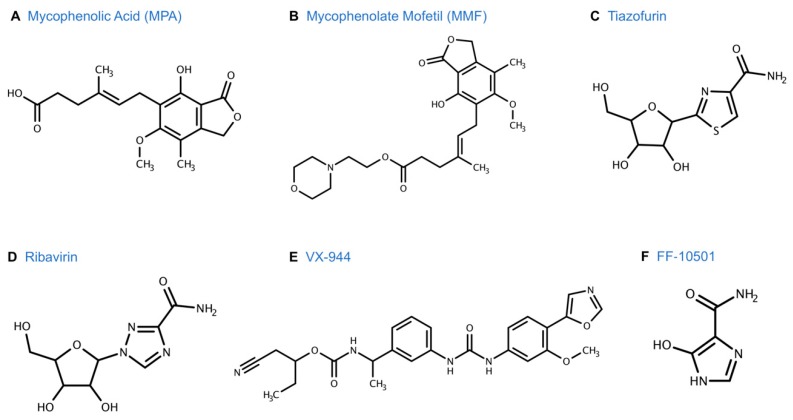
MPA and other IMPDH inhibitors and chemical structures.

The anti-tumor activity of MPA (Figure 4A) has been known since the late 1960s [59,62,63,64]. MPA was shown to suppress cell proliferation of leukemia, lymphoma, pancreatic cancer, non-small cell lung adenocarcinoma, and colon cancer cell lines [11]. MPA also induced differentiation or apoptosis of several cancer cell lines including breast [65], prostate [66], melanoma [67], leukemia [68], and neuroblastoma [69]. In 2004, based on the apoptotic properties of MMF (Figure 4B) noted by Dr. Takebe et al. [70], a phase I clinical trial was conducted using MMF in relapsed/refractory multiple myeloma [71]. Doses ranged from 1–5 g/day and were well tolerated even at the maximum dose of 5 g/day. There was also a significant correlation with the decrease of GTP levels in peripheral blood-derived mononuclear cells to levels of MPA measured in some patients who were deemed responders (partial response and stable disease). However, the other patients showed marginal changes in GTP levels. This suggested the potential for monitoring MMF activity in clinical use, but it remains unclear why peripheral blood GTP was only decreased in a subset of patients. 

In 2013, MPA activity in pancreatic ductal carcinoma (PDA) and its anti-angiogenic effects was tested using six patient-derived xenograft mouse models (PDX), followed by a pilot proof of concept study performed in resectable pancreatic cancer patients [72]. In the PDX study, one of the patient-derived PDA tumors showed a significant response to MMF treatment, with the tumor size decreasing to less than half (46%) compared to the vehicle control. Another PDA tumor showed a partial response, decreasing size to 75%, compared to the control. Interestingly, there are two PDA tumors that showed progression with MMF treatment (121% and 148% increase in tumor size). Since these MMF-treated PDA tumors decreased vascular endothelial growth factor (VEGF) synthesis and secretion, the results suggested that there is likely a genetic factor(s) or cellular context that renders the PDA tumor susceptible to MMF. In the clinical trial, 12 patients received MMF (6 with 1 g/day and 6 with 2 g/day) for 5–15 days before surgery, compared to 6 non-treated patients. However, no significant anti-angiogenic effect was observed in MMF-treated patients, in contrast to the result of the PDX mouse study. Based on the limited growth inhibition activity in mice and the marginal responses in patients, further clinical development of MMF in PDA was not recommended following the study. 

It is conceivable that the marginal in vivo anti-pancreatic cancer effect of MMF could be due to desmoplasia and stromal components outnumbering pancreatic tumor cells, which is a proposed cause of drug resistance in pancreatic cancer [73]. In addition, whether MPA accessed the PDA tumors in mice and human patients, and what drug concentration was achieved within the tumor, are unclear. Regardless, these studies can serve as benchmarks for future pharmacodynamic studies using MMF in human patients.

### 4.2. Long-Term Treatment Effect of MPA/MMF in Tissue-Transplanted Patients

Post-transplant malignancy is a well-recognized complication of transplantation with a three-fold to four-fold increase in the incidence of cancer in transplant patients compared to age-matched controls in the general population [74,75]. This is in part a consequence of chronic immunosuppression increasing the risk for viral infection and expansion, including oncogenic viruses (e.g., Epstein-Barr virus (EBV), Hepatitis B virus (HBV)). Nonmelanoma skin cancers and post-transplant lymphoproliferative disorders (PTLD) lymphoma are the most common malignancies observed in these patients [76]. Multiple studies have shown decreased incidence of PTLD, other malignancies, and risk of death when using MMF as a part of immunosuppression [77,78,79,80]. This could be explained by one or more of a variety of documented MPA actions including: blocking expansion of EBV infected B lymphocytes, anti-viral effects on HIV and Hepatitis, potentiation of other anti-viral agents [81], and its reported anti-tumor properties. If future research clarifies the benefit and the mechanism of suppression using MMF, this may substantially improve personalized post-transplant treatment.

### 4.3. Metabolism of MPA and Improved Routes of Delivery 

The major drawback of MPA as an anticancer agent is its dose-limiting GI toxicity. Most of the GI side effects are thought to be secondary to enterocytes toxicity [82]. MPA is extensively glucuronidated at the phenol group, which generates an inactive glucuronide that is quickly cleared by the kidney (Figure 3). Thus, the effective serum MPA concentration declines quickly in-vivo, which hampers the development of MPA-based anti-tumor therapies. However, substitutions of the phenolic hydroxyl and all other chemical modifications of MPA to avoid glucuronidation drastically reduce activity against IMPDH [83]. In recent years, a series of mycophenolic adenine nucleotides were developed and are known as “MAD” compounds (mycophenolic adenine dinucleotide analogue) [84,85,86]. While these analogues were not as potent as MPA, they were resistant to glucuronidation, which suggests that they might become lead compounds for further modification in the future.

To surmount MPA’s drawbacks as an anti-tumor drug, we have employed nano-technology to generate a biodegradable, MPA-integrated nanofiber [87]. The first generation MPA-fiber released and sustained an MPA concentration of about 10 μΜ in cell culture media [87] and suppressed growth of both the human glioblastoma cell line U87MG and patient-derived glioblastoma neuro-spheres [87]. A guanosine supplement reversed the inhibition, which suggests a specific effect of MPA on GTP. These results suggest that local MPA delivery, an approach that obviates gastrointestinal toxicity, increases the MPA stability and maintains local high concentrations of MPA, which may be an effective strategy for glioblastoma, an aggressive malignancy that recurs in nearly all patients and comes with a mortality of greater than 90% at five years [88]. Since the recurrent tumors predominantly appear adjacent (~2 cm) to the original lesion [89,90,91], continuous delivery of MPA to the region of resection has high potential to suppress glioblastoma recurrence. More research is needed to develop MPA delivery as well as other MPA analogues with improved potency, selectivity, and toxicity profiles with the retention of the inhibitory potential against IMPDH.

## 5. Other IMPDH Inhibitors

In addition to MPA, a different series of structurally distinct IMPDH inhibitors has been evaluated as potential anti-cancer drugs. Hematological malignancies, such as leukemia and multiple myeloma, are typically targeted for two primary reasons. First, IMPDH activity is 15–42-fold higher in leukemia cells compared to normal leukocytes [92] and IMPDH inhibition leads to depletion of guanine nucleotides and reduction of cell proliferation, selectively, in leukemia cells compared to bone marrow leukocytes [93,94]. Second, unlike solid tumors, it is easy to determine the extent of IMPDH inhibition in hematological malignancies by monitoring the reduction of GTP levels in blood and bone marrow specimens. A measured GTP level within the tumor serves as a biomarker and has assisted in accurate dose titration in clinical trials, as detailed below.

### 5.1. Tiazofurin Trials for Hematological Malignancy and Solid Tumors

Tiazofurin, a C-nucleoside (2-beta-D-ribofuranosylthiazole-4-carboxamide), was the first anti-tumor agent in the class of new IMPDH inhibitors. It was first synthesized in 1977 [95] as part of research efforts at ICN Pharmaceuticals, Inc. to develop new antiviral agents [96]. Tiazofurin (Figure 4C) is structurally related to the antiviral agent, ribavirin (Figure 4D). While tiazofurin exhibited weak antiviral activity, it was found to be effective against cancer cells [96]. This prompted further development of tiazofurin as an antineoplastic agent, and it was introduced in clinical trials in 1983 under the sponsorship of the National Cancer Institute (NCI) [97].

**Figure 5:**
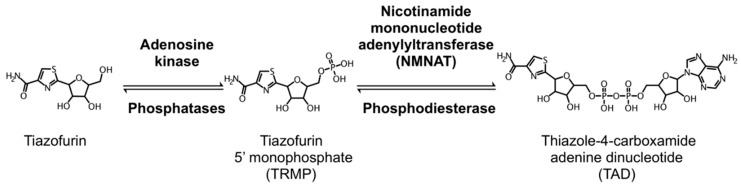
Intracellular metabolism of tiazofurin. Tiazofurin is a prodrug that is metabolized intracellularly in two steps to its active form TAD. TAD is an NAD analogue that inhibits IMPDH.

**Figure 6:**
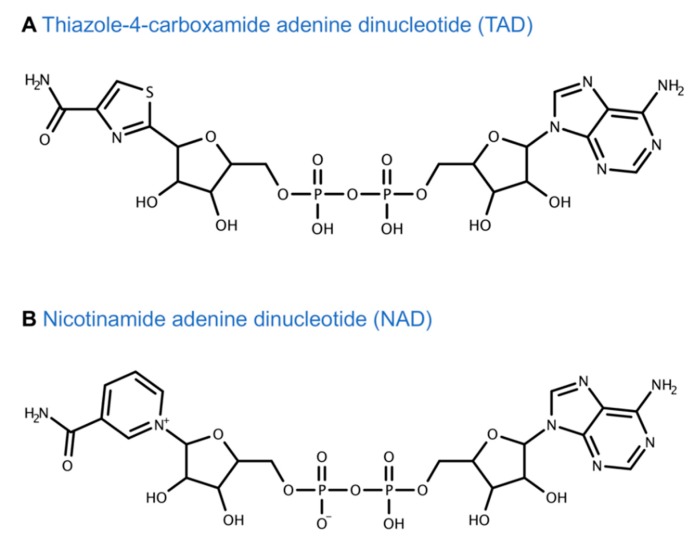
TAD and NAD chemical structures.

Tiazofurin is a prodrug and thus requires metabolic conversion intracellularly to its active metabolite thiazole-4-carboxamide adenine dinucleotide (TAD) in two sequential steps, as shown in Figure 5. TAD is an analogue of nicotinamide adenine dinucleotide (NAD) where nicotinamide is replaced by thiazole-4-carboxamide (Figure 6). TAD mimics NAD and interacts with the NAD cofactor binding domain of IMPDH 1 and 2 by acting as a non-competitive inhibitor [98,99,100,101]. Given that TAD is very similar to NAD (Figure 6), it is highly likely that the other NAD-dependent enzymes are affected by TAD. TAD is metabolically unstable and is degraded by nicotinamide mononucleotide adenylyltransferase (NMNATase), a phosphodiesterase. Resistance to tiazofurin is primarily associated with a decrease in NMNAT activity [92,101,102,103].

In vitro activity of tiazofurin was observed against many human cancer cell lines, including leukemia, colon, lung, ovarian, renal, breast, and melanoma [104,105,106,107,108,109]. Tiazofurin induced differentiation of the human promyelocytic leukemia cell line HL60 [110] and the erythroleukemia cell line K-562 [111]. In vivo cytotoxicity of tiazofurin was observed against several murine tumors, including Lewis lung carcinoma, hepatoma 3924A, and P388 and L1210 murine leukemias [112,113,114]. Tiazofurin was most efficacious against hematological malignancies. There was selective accumulation of TAD in leukemia cells compared to normal leukocytes [115,116]. Based on these findings, a phase I/II trial of tiazofurin in myeloid malignancies was conducted in 1987 that showed encouraging results especially in chronic myeloid leukemia in blast crisis (CML-BC) (Table 2). The overall response rate of 48% was very promising since the best reported objective response rate (ORR) in other Phase I and II trials between 1974 to 1982 ranged from 5.8% to 44% [117]. Despite significant toxicity, a subsequent successful phase II tiazofurin trial led in 2000 to orphan drug designation by the FDA for treatment of CML-BC.

One of the major limitations of therapy observed in these trials was the short duration of response and toxicity. There was rapid normalization of the white blood cell count and disappearance of blasts from the circulation within days of starting treatment. However, increased GTP levels were observed within a week of discontinuation of therapy, which lead to clinical relapse within three to four weeks [119]. Reinstitution of therapy initially led to a clinical response, but patients became refractory after a few cycles [103]. The second major limitation was the substantial toxicity profile of tiazofurin. This was anticipated to some extent because the close resemblance of tiazofurin to NAD would predict multiple targets besides IMPDH. Severe and life-threatening complications, including neurotoxicity, pleuropericarditis, and infections were observed in patients treated for longer than 15 days and with underlying comorbidities [117]. These were minimized by restricting the treatment duration, by administering the drug via one-hour daily infusions and promptly and effectively treating side effects [119,120,121]. After tiazofurin was granted orphan drug designation for treatment of CML-BC, a Phase III trial was planned. However, the development of imatinib and its FDA approval in 2001 revolutionized the treatment of Chronic Myelogenous Leukemia (CML) [122], and further development of tiazofurin for CML was halted.

Several phase I and phase II clinical trials of tiazofurin in advanced solid malignancies were conducted from 1983 to 1993 and are summarized in Table 3. Overall, despite promising results in vitro, tiazofurin showed minimal activity in solid tumors along with severe and unpredictable toxicities.

### 5.2. VX-944/AVN-944 and VX-497, Direct IMPDH Inhibitors

**Figure 7:**
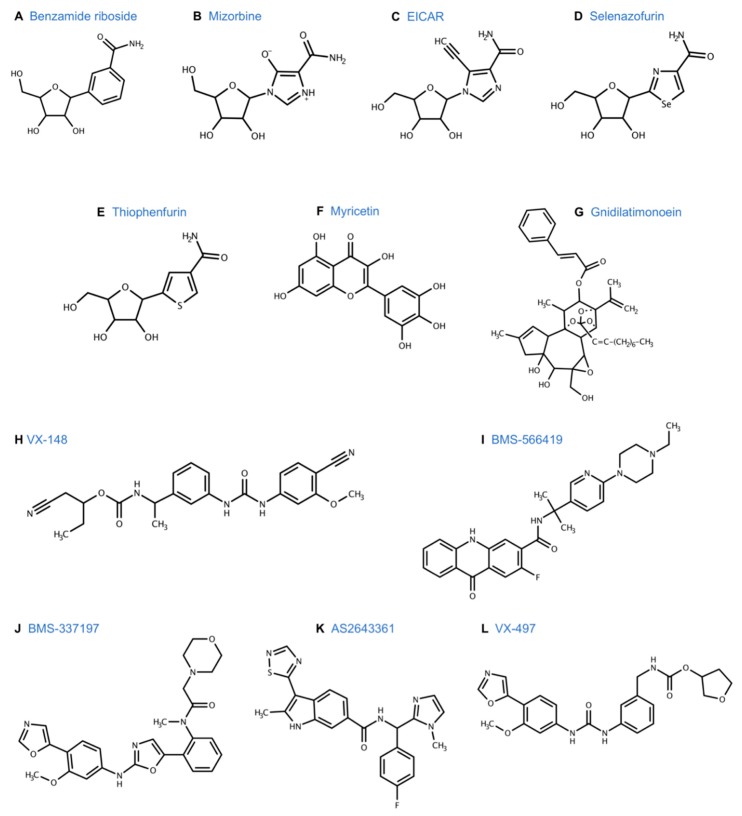
Other IMPDH inhibitors of chemical structures.

The development of tiazofurin as an anti-leukemia drug expanded the areas of clinical application of IMPDH inhibitors. Vertex Pharmaceuticals, Inc. developed a novel series of human IMPDH inhibitors that were structurally distinct from mycophenolic acid and nucleoside analogues [133]. Merimepodib (VX-497) (Figure 7L) was the lead compound developed in this series, and displayed immunosuppressive, anti-tumor, and anti-viral activity. As an immunosuppressive agent, it inhibited antibody production in vivo, as demonstrated by the murine plaque formation assay [134]. It had anti-proliferative activity against keratinocytes suggesting a possible role in the treatment of psoriasis [134]. VX-497 had broad spectrum anti-viral activity and was 10-100 times more potent than ribavirin against Hepatitis B, human cytomegalovirus, respiratory syncytial virus, and herpes simples virus [135]. More recently, VX-497 was found to be active against several globally emerging viruses like Zika virus and Ebola virus [136] reaching to Phase II trials for Hepatitis C and psoriasis [137], and displaying anti-tumor activity in vitro (Table 4).

A related compound, VX-944, was found to have broad anti-cancer properties in vitro and was investigated further [139] (Figure 4E). VX-944 is an orally bioavailable, small-molecule, non-competitive inhibitor of both human IMPDH1 and IMPDH2 [140]. VX-944 was developed using a structure-based drug design program and displayed a novel mode of interaction with IMPDH. Unlike tiazofurin, VX-944 does not require intracellular activation, which circumvents one of the mechanisms of resistance to tiazofurin. Since it is not a nucleoside/nucleotide analogue, VX-944 does not incorporate into the DNA/RNA and was predicted to work synergistically with other agents [141]. Preclinical studies showed that VX-944 was 3 to 40 times more potent than MPA in acute myeloid leukemia (AML) cell lines and was active against both FLT3 mutated and unmutated cells [139]. The efficacy of VX-944 was confirmed in a mouse model using a murine Ba/F3 pro-B cell line transformed with an oncogenic FLT3 mutant [142]. The mice treated with VX-944 had significantly longer median survival time compared to those treated with standard therapy. VX-944 suppressed proliferation of multiple myeloma cell lines, including drug-resistant cells [140], and several human cancer cell lines including colon, breast, lung, pancreatic, melanoma, and prostate [142].

Vertex entered into a licensing agreement with Avalon Pharmaceuticals in February 2005 for the development and commercialization of VX-944 in oncology as AVN-944 [143]. A phase I dose escalation study conducted in 2002 with 25 healthy male volunteers showed that AVN-944 was well tolerated [141]. In December 2005, a Phase I trial of AVN-944 in patients with advanced hematological malignancies commenced (ClinicalTrials.gov Identifier: NCT00273936). AVN-944 was well tolerated with no serious adverse events attributable to the drug. Additionally, 12 of 24 patients had a stable disease for 2 to 10 months [144]. Depletion of GTP pools, inhibition of IMPDH activity, and changes in gene expression were studied as biomarkers and demonstrated good correlation with a clinical response. Though more rigorous research is needed especially for the gene expression, these could be used to identify patients for Phase II trials. A phase II trial of AVN-944 in combination with gemcitabine, which is a current standard in pancreatic cancer treatment, commenced in June 2007 (ClinicalTrials.gov Identifier: NCT00493441), but the study was terminated in 2009 without reporting any results. Avalon pharmaceuticals was acquired by Clinical Data Inc. in 2009, which was acquired by Forest Labs in 2011. Since then, further clinical studies using AVN-944 have not been reported. The reason for the suspension is currently unclear.

### 5.3. FF-10501

**Figure 8:**
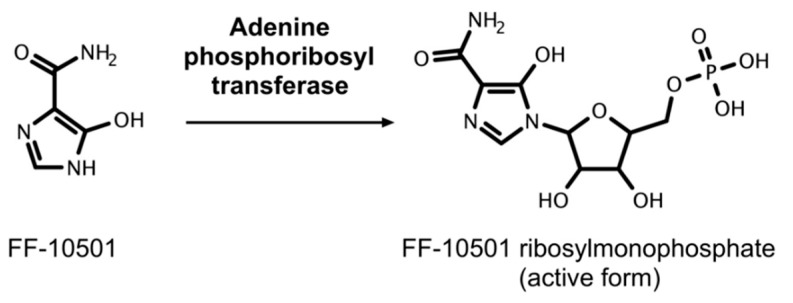
Activation of FF-10501. FF-10501 is metabolized intracellularly to its active form, FF-10501 ribosylmonophosphate (RMP), which inhibits IMPDH.

Fujifilm pharmaceuticals developed FF-10501 as part of its effort to develop new drugs for cancer treatment. FF-10501 is a purine-analogue antagonist and one of the most recently studied IMPDH inhibitors for the treatment of cancer (Figure 4F). It is an orally bioavailable, competitive, second generation inhibitor based on the previously studied SM-108 [145]. SM-108 was synthesized through chemical modification of the nucleoside mizoribine [146] and was found to be effective against several hematological malignancies in Phase I and II clinical trials conducted in Japan in the late 1980s [147,148,149,150]. FF-10501 is converted to its active form, FF-10501 ribosylmonophosphate (FF-10501RMP), intracellularly by using adenine phosphoribosyl transferase [151]. It reduces cell proliferation in a dose-dependent manner by inhibiting the production of guanine nucleotides (Figure 8). The efficacy of FF-10501 is dependent on the pathways that convert it to the active form as well as the salvage pathway to generate guanine nucleotides [145].

Pre-clinical studies showed the anti-leukemic effect of FF-10501 in multiple AML cell lines—MOLM13, SKM1, HL-60, U937, HEL, and OCI-AML3, including those that are resistant to hypomethylating agents [145,151]. The safety and efficacy of FF-10501 were tested in a phase I clinical trial, which is summarized in Table 5 [152,153]. A phase 2 study of FF-10501 in combination with azacitidine in patients with Myelodysplastic Syndrome (MDS) was initiated but has now been withdrawn without any enrollment [154]. Further studies to define the metabolic pathways that regulate sensitivity to FF-10501 and effective drug combinations may help increase enrollment for clinical trials.

Several new IMPDH inhibitors have been evaluated as anti-cancer drugs in pre-clinical studies. Many novel IMPDH inhibitors have been developed as antivirals and immunosuppressants. These are summarized in Table 6 and Table 7.

### 5.4. Future Directions for IMPDH Inhibitors as Anti-Tumor Drugs

Despite numerous efforts, many of which are summarized in this review, there remain no IMPDH inhibitors FDA approved for cancer treatment. The primary impediments continue to be (1) adverse effects upon high dose treatment, (2) highly variable responses, and (3) limited efficacy in cancers, such as pancreatic, in which IMPDH is not elevated. Overcoming these persistent challenges requires a greater fundamental understanding of the molecular features and roles of IMPDH enzymes. With this knowledge, inhibitors could be designed to preferentially target the tumor over normal cells. The remainder of this review summarizes recent efforts to understand the basic biology of IMPDH enzymes and GTP and how this might lead to advances in IMPDH inhibitor treatments.

## 6. Regulation of IMPDH by GTP

**Figure 9:**
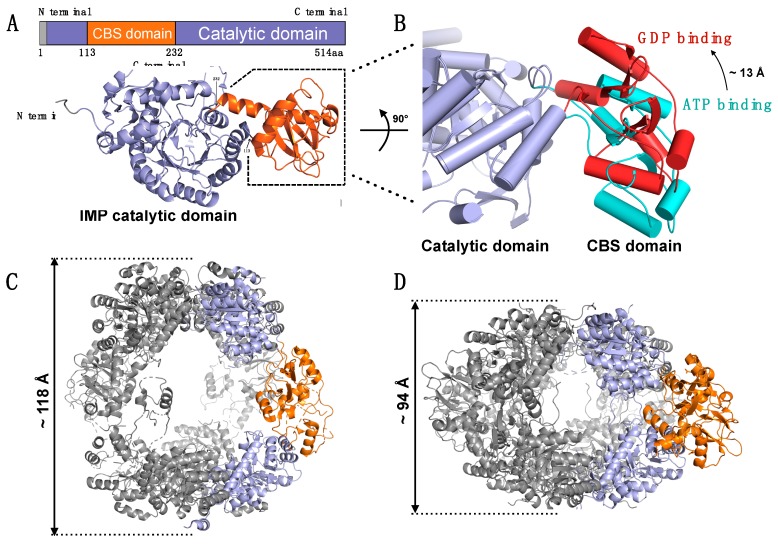
Schematics of IMPDH structures. (**A**) Schematic representation of the human IMPDH2 protein (upper) and monomer of human IMPDH2 structure (lower) (PDB ID: 6i0m). IMPDH structure is shown in cartoons while α-helixes are shown in a coiled model. The CBS domain is colored orange, and the MPDH catalytic domain is shown in light purple. (**B**) Structural changes of the CBS domain upon ATP (Cyan) and GDP (Red) binding in monomeric IMPDH from *Ashbya gossypii*. Superposed ATP binding (PDB ID: 5mcp) and GDP binding (PDB ID: 4z87) by using Cα overlap and 243 aa was aligned. A-helixes were shown in a cylindrical model. The CBS domain rotated toward an IMPDH catalytic domain (light blue) significantly when GTP binds to the CBS domain, compared with ATP binding. (**C**,**D**) Different octameric forms between ATP binding (**C**) and GTP binding (**D**). Two monomers of octameric IMPDH (Gray) are colored orange (CBS domain) and light purple (catalytic domain). The approximate longitudinal dimensions of the octamers are indicated on their side. Comparing to ATP binding (**C**), the interaction changes between CBS domains upon GTP binding made the octameric structure of human IMPDH2 (**D**, PDB ID: 6i0o) more compact. Since no ATP-bound structure of human IMPDH has been determined, IMPDH from *Ashbya gossypii* (PDB ID: 5MCP) is used as the ATP binding model.

IMPDH1 and IMPDH2 share more than 80% identity at the amino acid level and are primarily found as a tetramer in vitro [216]. Both are comprised of two domains: the (α/β)_8_ barrel (also known as a TIM barrel) core containing the active site, and the disordered 120-residue subdomain, which consists of two cystathionine-β-synthase (CBS) domains (Figure 9A) [217,218]. The CBS domain is a ~60 amino acid domain discovered in 1997 by Dr. Alex Bateman and is found in a variety of proteins, including IMPDH, cystathionine-β-synthase, chloride channels, and AMP-activated protein kinase (AMPK) [219]. CBS domains are typically found as tandem repeats (also known as a Bateman domain) that can adopt a globular protein structure via intramolecular folding [219,220]. The function of the CBS domain is diverse and can range from a binding site for allosteric regulation to regulatory protein binding to multimerization [221,222]. The CBS domains of IMPDH, however, have little known function. Deletion of the subdomain has no effect on enzymatic activity in vitro [223]. Despite this, the CBS domains of both IMPDH1 and IMPDH2 are suspected to be involved in regulating enzymatic activity.

Supporting evidence of this concept was reported by Dr. Buey et al. with the discovery of GTP-binding sites within the CBS domains of fungal IMPDH, which lead to octamerization of the enzyme and subsequent inhibition of activity [224]. The inhibitory effect of GTP was also observed in human IMPDH1 and IMPDH2 [224]. This data suggests a form of negative feedback involving IMPDH, where the end-product GTP inhibits the biosynthesis of GTP through inhibition of IMPDH. In a subsequent series of studies, Dr. Buey’s group further revealed that the CBS domain of IMPDH can bind to three GTP/GDP molecules, including two that bind to a similar pocket to ATP. However, the third GDP molecule binds to the loop between the second CBS domain and the catalytic domain. GDP binding at this site causes rotation in the CBS domain and structural rearrangement of the catalytic domain (Figure 9B). This structural change significantly affects the enzyme conformation, which forms an inhibited conformation, as compared to the ATP-induced active form [225]. Thus, interaction with GTP/GDP decreases the flexibility of the CBS domain, and the activity of IMPDH is suppressed by forming a compact structure (Figure 9C,D).

## 7. Dynamic Feature of IMPDH—Macrostructural Formation

**Figure 10:**
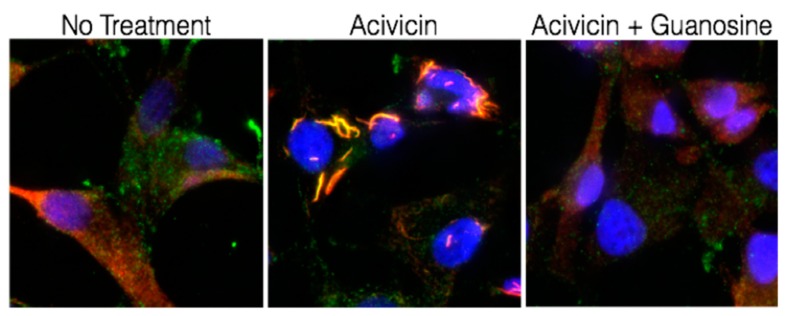
IMPDH Ring and Rod (RR) structure is responsive to GTP concentration. A total of 100 μM Acivicin (GMP synthase inhibitor) treatment for 6 hours decreased cellular GTP levels (data not shown) and induced RR structure formation in U87MG cells, which was rescued by 2 hours pre-treatment with 100 μM guanosine that increased cellular GTP (data not shown).

Interestingly, IMPDH is one of several metabolic enzymes that organize into large filaments [226,227,228,229,230,231]. Multiple groups, including ours, have found the localization of IMPDH1 and IMPDH2 are influenced by intracellular GTP concentration, which forms self-assembled, macroscale assemblies, called Ring and Rod (RR) structures or Cytoophidia (Figure 10) [232,233,234,235]. In most cell types under steady state growing conditions, IMPDH isozymes localize primarily to the cytoplasm. However, under purine-depleted conditions, primarily when GTP biosynthesis is inhibited, IMPDH organizes into these RR structures most prominently found in the cytoplasm [233,234,236,237]. The RR structure does not colocalize with any organelles and does not associate with actin or tubulin [232,234].

The effect of the RR structure formation on enzymatic activity remains controversial, with evidence supporting both increased activity [238] and inhibition [239]. Potential IMPDH isotype-specific differences and the possibility of mixed-isoform structures further hinder progress in this area. One common theme for RR structure formation appears to be decreased intracellular GTP levels, with subsequent dissociation of RR following guanosine treatment, which is a metabolite of the salvage pathway, that restores GTP concentration to normal or supraphysiological levels [233,234,236,237,239]. According to the recent research by Dr. Fernandez-Justel et al. [239], purified human IMPDH could assemble into RR formation without nucleotide treatment in vitro. They showed that the RR form is catalytically active in vitro, and GTP/GDP within the CBS domains could depolymerize the RR conformation to suppress IMPDH activity. Moreover, the RR form is more resistant to GTP inhibition by enzyme assay analysis in vitro [240].

## 8. IMPDH Immunohistochemical Analysis May Report the Metabolic Status of Tumors

Taking these discoveries into consideration, we propose that pathological analysis of IMPDH in tumors has a high potential to assess the GTP demand and metabolic status of tumors. We favor the model that RR structure-positive tumors would be more sensitive to IMPDH inhibitors because of their high demand for GTP. If this were the case, it is possible that we may increase the anti-tumor efficacy of IMPDH inhibitors by selecting patients based on their RR structure status. Additionally, it would be of interest to develop compounds that specifically target the RR structure formation. Alternatively, it is possible that RR structure formation may alter drug accessibility to IMPDH or sensitivity to IMPDH inhibitors. The physiological relevance of the RR structure remains to be tested. Additionally, several studies have shown the generation of autoantibodies for RR structures in patient serum, particularly in hepatitis C patients treated with interferon α and ribavirin. Although auto-antibody formation in human cancer patients exhibiting RR structure-positive tumors has not been studied, theoretically this could provide a noninvasive predictor of a patient’s response to IMPDH inhibitors.

## 9. Potential Biomarker for the Anti-Tumor Effect of IMPDH Inhibitors in the Target Tumor

As stressed throughout this review, the key challenge is increasing both the specificity, efficacy, and kinds of cancer susceptible to IMPDH inhibitors without incurring unacceptable side effects. Better pharmacodynamic markers would be helpful to this end. For instance, in most treated patients, it is not known if effective concentrations of the IMPDH inhibitor are achieved within the tumors due to a lack of biomarkers for IMPDH inhibition. To measure the pharmacodynamics of IMPDH inhibitor penetrance in tumors as well as guanine nucleotides ideally requires a direct measurement. Recent advances in mass spectrometry technology to measure metabolites may solve this challenge. A caveat of this approach is that mass spectrometric analysis is not conventional in hospitals and would require days or weeks to receive results. Additionally, it may require relatively large tissue samples, and nucleotides rapidly decay during extraction.

Dr. Beverly Mitchell’s group, a leader in the IMPDH research field, has reported that inhibition of IMPDH results in nucleolar stress responses, p53 activation, and the dichotomic change of subcellular localization of nucleolar proteins, such as nucleolin and nucleostemin [241]. Thus, the detection of p53 levels and its downstream targets, such as monitoring p21^CIP1/WAF1^, as well as subcellular localization of nucleostemin and nucleolin could be an indirect means to assess the efficacy of IMPDH inhibitors in the target tumors. Furthermore, our recent study showed that treatment with IMPDH inhibitor, MPA, led to significant nucleolar stress responses, p53 activation, and decreased nucleolar size in GBM, but not in primary cells [2]. Moreover, we demonstrated that IMPDH2 upregulation is required to increase GTP concentration that can serve as a reservoir of “feeder” GTP to sustain the needs of high activity of RNA Pol I and III for rRNA and tRNA synthesis, respectively [2]. To assess the effect of IMPDH inhibition, one would need simple conventional methods, such as Q-PCR or immunohistochemical staining of tumor samples, to indirectly determine IMPDH inhibitor penetrance into the tumor. With further verification, these biomarkers could be critical, powerful tools for future clinical trials, and also be useful in preclinical settings during the development and testing of novel IMPDH inhibitors in tumor mouse models.

## 10. Conclusions

Recent advances in our basic understanding of the role of IMPDH in normal physiology and cancer justify a reappraisal of the potential efficacy of IMPDH inhibitors. Future studies could focus on developing modified IMPDH inhibitors with more diverse structures and different binding modes of enzyme inhibition to hopefully provide additional guidance for clinical trial design that would, ultimately, result in the use of IMPDH inhibitors for the treatment of cancer. The development of biomarkers should significantly and critically improve tumor selectivity and predict patient response to IMPDH inhibitors. In addition, with the recent advent of checkpoint inhibitors that rely upon the patient’s immune system to attack and destroy the tumor [242], the possibility of combining these approaches with IMPDH inhibitors that could ameliorate adverse-effects of the checkpoint inhibitor as well as modulating the tumor immune microenvironment are important areas to explore. Future research clarifying these points may revolutionize the use of IMPDH inhibitor as a highly potent anti-tumor drug as has been suggested since the 1960s.

## Figures and Tables

**Table 1:** Labeled and off-labeled indications of MMF.

Indication	Studies
**Labeled (FDA Approved) Indications**	**Studies that led to FDA approvals**
Renal transplant	Sollinger 1995 [24], Grinyo 1995 [25], Keown 1996 [26]
Liver transplant	Eckhoff 1998 [27], Wiesner 2005 [28], Nashan 2009 [29]
Cardiac transplant	Eisen 2005 [30], Kobashigawa 2006 [31], Kaczarek 2013 [32], Andreassen 2014 [33]
**Off-Label Use**	**Studies supporting off-label use**
Lung transplant	Treede 2001 [34], Zuckermann 2003 [35], Speich 2010 [36]
Pancreatic transplant	Ricart 2012 [37], Descourouez 2018 [38]
Refractory acute graft-versus-host disease	Alousi 2009 [39]
Refractory chronic graft-versus-host disease	Wolff 2010 [40]
Prevention of graft-versus-host disease	Sabry 2009 [41]
Aplastic anemia	Scheinberg 2006 [42]
Autoimmune hepatitis, first line	Zachou 2016 [43]
Refractory autoimmune hepatitis	Manns 2010 [44]
Lupus nephritis	Contreras 2004 [45], Ong 2005 [46], Dooley 2011 [47], Hahn 2012 [48]
Myasthenia gravis	Meriggiolo 2003 [49], Sanders 2016 [50], Sieb 2014 [51]
Psoriasis	Menter 2009 [52]
Systemic sclerosis	Gerbino 2008 [53], Derk 2009 [54], Le 2011 [55], Mendoza 2012 [56], Tashkin 2016 [57], Herrick 2017 [58]

**Table 2:** Summary of clinical trials of Tiazofurin for hematological malignancy.

Phase	Study Population	Dose	Clinical Response	References
I/II	Relapsed/refractory AML, CML-BC, and MDS. n = 27	Biochemically directed protocol. Starting dose 2200 mg/m^2^ daily, dose escalated based on IMPDH and GTP levels in the leukemic cells	Complete response (CR) 20% Objective response rate (ORR) 48%	[117,118,119]
II	CML-BC n = 6	Started at 2200 mg/m^2^ daily for 10 days and escalated based on hematological and biochemical response.	Objective response rate (ORR) 100% but no complete response (CR)	[120]

**Table 3:** Summary of clinical trials of Tiazofurin for solid malignancy.

Phase	Study Population	Dose	Clinical Response	References
I	Advanced solid malignancies	Maximum tolerated dose varied between studies	Response reported with only one trial Maroun et al. reported 12 of 25 patients had stable disease, including one patient with anaplastic astrocytoma who was in remission for 50 months.	[123,124,125,126,127,128]
II	Glioma	1100 to 1375 mg/m^2^ IV daily for five days	Five of 16 patients had stable disease for a median of 75 days, but no responses seen.	[129,130,131,132]

**Table 4:** Summary of VX-497 as an anti-viral reagent.

IMPDH Inhibitor	Mechanism of Action	Study Results
VX-497 (Merimepodib) (*S*)-*N*-3-[3-(3-methoxy-4-oxazol-5-yl-phenyl)-ureido]-benzyl-carbamic acid tetrahydrofuran-3-yl-ester (Figure 7L)	VX-497 is a non-nucleoside, orally bioavailable, selective, reversible, uncompetitive inhibitor of IMPDH, which was developed by Vertex Pharmaceuticals [134].	Clinical studies VX-497 was efficacious as monotherapy for Hepatitis C in combination with interferon-alpha in treatment naïve patients [138]. However, phase II trials in patients with genotype 1 chronic hepatitis C, who were non responders to standard treatment, showed mixed results.

**Table 5:** Summary of a clinical trial of FF-10501.

Phase	Study Population	Dose	Clinical Response	Toxicity	References
I	Relapsed/refractory AML and MDS n = 37	Escalating doses from 50–500 mg/m^2^. Recommended phase II dose 400 mg/m^2^ for 21 days every 28-day cycle.	Response observed in 4 of 37 patients	Well tolerated, frequently Grade 1–2	[152,153]

**Table 6:** Summary of preclinical studies of other IMPDH inhibitors as antitumor agents.

IMPDH Inhibitor	Mechanism of Action	Study Results
Reversible nucleoside inhibitors		
Benzamide riboside (BR) 3-(1-Deoxyribofuranosyl) benzamide (Figure 7A)	Benzamide riboside (BR) was first synthesized in 1992 [155]. Similar to tiazofurin, BR, is converted to its active metabolite, BAD (benzamide adenine dinucleotide) intracellularly via NMNAT. BAD is proposed as a dual inhibitor of IMPDH and NAD kinase. IMPDH inhibition leads to depletion of guanine nucleotides and halts DNA/RNA synthesis [156,157]. NAD kinase inhibition leads to decreased levels of NADPH. Low NADP^+^ and NADPH levels lead to instability and lower levels of dihydrofolate reductase [158].	BR was more cytotoxic than tiazofurin in a broad panel of human cancer cell lines, including leukemia, lung, colon, CNS, melanoma, ovarian, and renal cell carcinoma [104,159,160]. CNS cell lines showed selective sensitivity to BR. BR was 3-10 times more cytotoxic than tiazofurin against leukemia [104]. In vivo, BR prolonged survival of a mouse model with murine leukemia L1210 [161] but caused significant skeletal muscle toxicity [162]. BR induced apoptosis in the VX2 model of liver cancer in rabbits via hepatic artery infusion [163]. Mouse model of LX-1 human small cell lung carcinoma was relatively refractory to treatment with BR in vivo and the high doses required for anti-tumor effect lead to significant morbidity and mortality [162]. The clinical application of BR was limited by its toxicity profile.
Mizoribine (MZR) (INN, trade name Bredinin) 5-hydroxy-1-β-D-ribofuranosyl-1H-imidazole-4-carboxamide (Figure 7B)	An imidazole nucleoside isolated from *Eupenicillium brefeldianum*, mizoribine (MZR) is metabolized to MZR-5’-monophosphate (MZRP) by adenosine kinase. MZRP, the active metabolite, inhibits IMPDH and guanosine monophosphate synthetase, which are sequential enzymes in the de novo pathway. Therefore, MZR completely inhibits the synthesis of guanine nucleotides [164,165,166]. MZR selectively inhibits lymphocyte proliferation, thereby inhibiting both humoral and cellular immunity [167,168].	MZR was originally isolated as an antibiotic with activity against *Candida albicans* [167] but was subsequently found to have potent immunosuppressive activity [169]. Preclinical Early pre-clinical studies reported that MZR was not active against mice inoculated with Ehrlich and P388 tumor cells and had a minimal life prolonging effect on mice inoculated with L1210 leukemia cells [167]. However, more recently, MZR was found to produce a marked anti-leukemic response and increased survival in mice inoculated with resistant acute lymphoblastic leukemia with NT5C2+/R367Q mutation [170]. The expression status of adenosine kinase dramatically affects the efficacy of MZR [157]. Further preclinical studies are needed to better define the role of MZR in leukemia. Clinical MZR is currently used as an immunosuppressive drug. It has a favorable adverse effect profile and is usually used in combination with other drugs. It has been approved in Japan to prevent rejection after renal transplantation (1984), lupus nephritis (1990), rheumatoid arthritis (1992), and nephritic syndrome (1995) [171]. The use of MZR is being investigated in other nephropathies [172], pemphigus vulgaris [173], and polymyalgia rheumatica [174].
Ribavirin 1-β-D-ribofuranosyl-1,2,4-triazole-3-carboxamide (Figure 4D)	Ribavirin is a guanosine analogue that is phosphorylated intracellularly to ribavirin-5-monophosphate, which inhibits IMPDH [175]. Ribavirin has broad spectrum antiviral activity [176]. It exerts antitumor activity through inhibition of IMPDH, eukaryotic translation initiation factor 4E (eIF4E), and histone methyltransferase, Enhancer of Zeste Homolog 2 (EZH2) [177,178].	Pre-clinical studies have shown that ribavirin inhibits the proliferation of several tumor types including malignant glioma [177], acute myeloid leukemia [179], acute lymphoblastic leukemia [180], esophageal [181], colon, cervical [182], breast [183], and prostate cancer [184]. Clinical studies Phase I/II trials are underway for assessing the use of ribavirin in various cancers including head and neck cancer, mantle cell, and follicular lymphoma [185,186,187]. Ribavirin has been approved by the FDA as an inhaled agent for respiratory syncytial virus [188] and in combination with interferon-alpha for the treatment of chronic hepatitis C [189].
EICAR 5-ethynyl-1- β -D-ribofuranosylimidazole-4-carboxamide (Figure 7C)	Imidazole derivative of ribavirin, EICAR is metabolized intracellularly via adenosine kinase to EICAR 5’-monophosphate, which inhibits IMPDH [190].	EICAR had broad antiviral activity, which was 10-100 fold greater than ribavirin [191]. It was cytotoxic to several human cancer cell lines in vitro and murine leukemia L1210 and P388 in vivo [192].
Selenazofurin 2- β -D-ribofuranosylselenazole-4-carboxamide (Figure 7D)	Selenium analogue of tiazofurin, selenazofurin is converted to its active metabolite, selenazole-4-carboxamide adenine dinucleotide (SAD) intracellularly, via NMNAT. SAD is a NAD analogue and inhibits IMPDH [193,194].	As an antitumor agent, selenazofurin was found to be 5–10 fold more potent compared to tiazofurin in several in vitro studies [194]. It had broad antiviral activity [195] and was synergistic in combination with ribavirin [196].
Thiophenfurin 5-β-D-ribofuranosylthiophene-3-carboxamide (Figure 7E)	Thiophene analogue of tiazofurin, it is converted intracellularly to thiophene-3-carboxamide adenine dinucleotide (TFAD), a NAD analogue, which inhibits IMPDH [197].	In vitro studies showed that thiophenfurine was cytotoxic toward several cancer cell lines, including human promyelocytic leukemia HL-60, human colon adenocarcinoma LoVo, and B16 melanoma at similar concentrations as tiazofurin [197].
Flavonoids		
Myricetin 3,5,7-trihydroxy-2-(3,4,5-trihydroxyphenyl)-4-chromenone (Figure 7F)	Myricetin is a dietary flavonoid found in berries and vegetables. It causes cell cycle arrest and apoptosis through various mechanisms, including inhibition of tumorigenic kinases [198], which increases mitochondrial apoptotic pathways, reactive oxygen species, and IMPDH inhibition [199].	Myricetin has extensive biological activity, including anti-viral, anti-inflammatory, and anti-cancer [200]. In vitro studies have shown that myricetin has anti-leukemia effect on K562 cell lines through IMPDH inhibition [199]. It is cytotoxic to several other human cancer cell lines like colon [201], ovarian [202], prostate [203], breast [204], and thyroid [205] cancer cell lines by targeting various pathways.
Diterpene ester		
Gnidilatimonoein (Gn) (Figure 7G)	Diterpene ester isolated from the leaves of *Daphne mucronata*, Gn exerts anti-neoplastic activity through inhibition of IMPDH [206].	In vitro studies have shown that Gn has antiproliferative activity against several human cancer cell lines and induced differentiation in the HL-60 human leukemia cell line [207].

**Table 7:** Summary of IMPDH inhibitors as immunosuppressants.

IMPDH Inhibitor	Mechanism of Action	Study Results
Mizoribine	See Table 5.	
VX-148 1-cyanobutan-2-yl *N*-[(1*S*)-1-[3-[(4-cyano-3-methoxyphenyl) carbamoyl amino] phenyl] ethyl] carbamate (Figure 7H)	VX-148 noncompetitively inhibits IMPDH by binding to the NAD cofactor binding site. It is an orally bioavailable small molecule that was developed by structural modification of VX-497 by Vertex Pharmaceuticals [208].	VX-148 was found to have in vivo and in vitro immunosuppressive activity similar to MPA but with less cytotoxicity [208]. Vertex Pharmaceuticals selected it as its lead drug development candidate for autoimmune diseases [134]. VX-148 has been evaluated in a Phase II trial in moderate to severe psoriasis in 2004. It was well tolerated. The most frequent adverse events were diarrhea and itching. It showed a statistically significant clinical activity with a response rate of 18% compared to a placebo [209,210].
BMS-566419 N-(1-(6-(4-Ethyl-1-piperazinyl)-3-pyridinyl)-1-methylethyl)-2-fluoro-9,10-dihydro-9-oxo-3-acridinecarboxamide (Figure 7I)	Acridone based derivative of VX-497, BMS-566419 is an orally bioavailable IMPDH inhibitor developed in 2007 [211].	In vitro studies demonstrated the anti-proliferative activity of BMS-566419 on immune cells. Preclinical studies showed that it was efficacious in the murine model of rheumatoid arthritis and prevented cardiac allograft rejection with less GI toxicity compared to MMF [211,212].
BMS-337197 N-[2-[2-(3-methoxy-4-oxazol-5-yl-anilino) oxazol-5-yl] phenyl]-N-methyl-2-morpholino-acetamide (Figure 7J)	2-aminooxazole derivative of VX-497, BMS-337197 is an orally bioavailable, uncompetitive inhibitor of IMPDH [213].	Preclinical studies showed that BMS-337197 had potent immunosuppressive activity. It inhibited antibody production in mice and was efficacious as an anti-arthritis drug in a murine model of rheumatoid arthritis [214].
AS2643361 N-((4-fluorophenyl) (1-methyl-1H-imidazol-2-yl) methyl)- 2-methyl-3-(1,2,4-thiadiazol-5-yl)-1H-indole-6-carboxamide (Figure 7K)	An indole derivative of MMF developed from the Astellas compound library, AS2643361 is an orally bioavailable IMPDH inhibitor [215]. In vitro, it has similar inhibitory activity as mycophenolate to inhibit IMPDH.	AS2643361 had lower serum protein binding activity. In vivo, it showed higher potency and less toxicity than MMF as an immunosuppressant. It prevented cardiac allograft rejection in a murine model [215].
